# Poster Session I - A29 NUTRIENT-SENSING BY INTESTINAL TUFT CELLS: IMPLICATIONS FOR INFLAMMATORY BOWEL DISEASE

**DOI:** 10.1093/jcag/gwaf042.029

**Published:** 2026-02-13

**Authors:** A Kirkwood, F Larsen, S Asfaha, V Lu

**Affiliations:** Physiology and Pharmacology, Western University, London, ON, Canada; Physiology and Pharmacology, Western University, London, ON, Canada; Physiology and Pharmacology, Western University, London, ON, Canada; Physiology and Pharmacology, Western University, London, ON, Canada

## Abstract

**Background:**

Inflammatory bowel disease (IBD), encompassing Crohn’s disease and ulcerative colitis, refers to chronic inflammatory conditions of the intestines that are characterized by intestinal barrier dysfunction. While the exact cause of this loss of gut barrier function in IBD is unknown, recent studies suggest that a rare subset of secretory intestinal epithelial cells, the tuft cells (TC), may have a protective role in maintaining the gut barrier.

Only recently have we gained insight into the function of this class of cells. Upon activation, TCs secrete interleukin-25 (IL-25), which triggers a type 2 immune response that results in extensive tuft and goblet cell hyperplasia to restore the gut barrier. Notably, external nutrients such as succinate have been shown to activate TCs and increase their abundance. This raises an intriguing opportunity to target the intestinal TC population and restore the gut barrier through dietary approaches. However, other nutrients capable of stimulating TCs and their responsiveness to gut bacterial metabolites remain unknown.

**Aims:**

This study aims to identify nutrient-sensitive receptors expressed by intestinal TCs and to characterize the signaling pathways through which they influence IL-25 secretion and gut barrier maintenance.

**Methods:**

Transcriptomic databases were analyzed to identify candidate nutrient-sensing receptors selectively expressed in TCs. Functional studies were performed using intestinal organoids derived from *Dclk1*-CreERT2 × *Rosa26*-tdTomato mice, in which TCs were stimulated with nutrients or synthetic ligands. IL-25 secretion was quantified by ELISA, while intracellular signaling mechanisms downstream of receptor activation were assessed through Fura-2 calcium imaging.

**Results:**

Transcriptomic profiling identified selective expression of *Sucnr1* (succinate receptor 1) and *Ffar3* (free fatty acid receptor 3) in intestinal TCs, along with additional candidate receptors (*Gprc5c, Grik3, Vmn2r26*). Functional assays revealed that stimulation with succinate, butyrate (an FFAR3 ligand), or the synthetic FFAR3-selective agonist AR420626 did not increase IL-25 secretion.

**Conclusions:**

This study seeks to advance our current understanding of TC physiology and identify novel therapeutic targets to restore gut barrier function in IBD patients. While acute succinate and butyrate treatment did not elicit IL-25 secretion, ongoing studies are exploring additional nutrient ligands and receptors that may underlie TC-mediated epithelial protection and offer novel therapeutic avenues for IBD.

**Funding Agencies:**

NSERC, Children’s Health Research Institute, London, ON

